# Exercise as Modulator of Brain-Derived Neurotrophic Factor in Adolescents: A Systematic Review of Randomized Controlled Trials

**DOI:** 10.3390/sports13080253

**Published:** 2025-08-01

**Authors:** Markel Rico-González, Daniel González-Devesa, Carlos D. Gómez-Carmona, Adrián Moreno-Villanueva

**Affiliations:** 1 Department of Didactics of Music, Plastic and Body Expression, University of the Basque Country, UPV-EHU, 48940 Leioa, Spain; markel.rico@ehu.eus; 2 Research Group on Physical Activity, Education, and Health (GIAFES), Catholic University of Ávila, 05005 Ávila, Spain; 3 Research Group in Training, Physical Activity and Sports Performance (ENFYRED), Department of Music, Plastic and Body Expression, University of Zaragoza, 44003 Teruel, Spain; 4 Research Group in Training Optimization and Sports Performance (GOERD), University of Extremadura, 10005 Caceres, Spain; 5 BioVetMed & SportSci Research Group, University of Murcia, 30001 Murcia, Spain; 6 Faculty of Health Science, University Isabel I, 09003 Burgos, Spain; adrian.moreno@ui1.es

**Keywords:** BDNF, blood, biomarker, learning, plasticity

## Abstract

Adolescence represents a critical period of neurodevelopment during which brain-derived neurotrophic factor (BDNF) plays a fundamental role in neuronal survival and synaptic plasticity. While exercise-BDNF relationships are well-documented in adults, evidence in adolescents remains limited and inconsistent. This systematic review examined the effects of exercise modalities on circulating BDNF concentrations in adolescent populations. A systematic search was conducted following PRISMA guidelines across multiple databases (FECYT, PubMed, SPORTDiscus, ProQuest Central, SCOPUS, Cochrane Library) through June 2025. Inclusion criteria comprised adolescents, exercise interventions, BDNF outcomes, and randomized controlled trial design. Methodological quality was assessed using the PEDro scale. From 130 initially identified articles, 8 randomized controlled trials were included, with 4 rated as excellent and the other 4 as good quality. Exercise modalities included aerobic, resistance, concurrent, high-intensity interval training, Taekwondo, and whole-body vibration, with durations ranging 6–24 weeks. Four studies demonstrated statistically significant BDNF increases following exercise interventions, four showed no significant changes, and one reported transient reduction. Positive outcomes occurred primarily with vigorous-intensity protocols implemented for a minimum of six weeks. Meta-analysis was not feasible due to high heterogeneity in populations, interventions, and control conditions. Moreover, variation in post-exercise sampling timing further limited comparability of BDNF results. Future research should standardize protocols and examine longer interventions to clarify exercise-BDNF relationships in adolescents.

## 1. Introduction

Adolescence represents a critical period of neurodevelopment characterized by extensive neuroplastic changes, particularly in prefrontal cortical regions that govern executive functions essential for cognitive performance and academic achievement [1]. During this developmental stage, the brain exhibits heightened sensitivity to environmental influences, making it an optimal window for interventions aimed at enhancing neural health and cognitive capacity. Physical exercise has emerged as a promising modulator of brain function and development, with mounting evidence suggesting its potential to promote neuroplasticity through various molecular mechanisms [2,3].

Brain-derived neurotrophic factor (BDNF) is the most abundant neurotrophin in the mammalian brain, with high concentrations found in the hippocampus, cerebral cortex, hypothalamus, and cerebellum [4,5]. This protein plays a fundamental role in neuronal survival, differentiation, and synaptic plasticity, serving as a critical mediator of learning, memory formation, and cognitive function [6,7]. BDNF exerts its effects through binding to the tropomyosin receptor kinase B (TrkB), initiating signaling cascades that promote neurogenesis, dendritic branching, and synaptic strengthening [8]. Importantly, BDNF can cross the blood-brain barrier bidirectionally, allowing peripheral concentrations in serum and plasma to serve as accessible biomarkers of central nervous system BDNF activity [9].

The relationship between physical exercise and BDNF has been extensively documented in adult populations, with systematic reviews and meta-analyses by Szuhany et al. [10] and Dinoff et al. [11] demonstrating that both acute and chronic exercise interventions can modulate peripheral BDNF concentrations, with moderate-to-vigorous intensity aerobic exercise producing the most robust BDNF elevations [12,13]. However, the evidence regarding exercise-induced BDNF modulation in adolescent populations (12–18 years) remains substantially more limited and inconsistent, with only a handful of studies specifically examining this relationship in pediatric populations.

While systematic reviews by Donnelly et al. [14] and Carson et al. [15] (examining early childhood populations) have established clear associations between physical activity and cognitive development in younger populations, and recent meta-analyses by Menezes-Junior et al. [16] and Acevedo et al. [17] have demonstrated exercise effects on neurocognitive outcomes in children and adolescents, the specific neurobiological mechanisms underlying these relationships, particularly the role of BDNF, require further elucidation and actualization. The few studies that have examined BDNF responses to exercise in adolescents have yielded mixed results, with some investigations reporting significant increases following aerobic training protocols [18,19], while others have found no meaningful changes across various exercise modalities [20,21].

Recent systematic reviews have highlighted the need for updated analyses focusing specifically on adolescent populations and higher-quality study designs [22,23]. Several factors unique to adolescent populations may contribute to heterogeneity in findings, including ongoing neurodevelopmental processes, hormonal fluctuations, varying baseline fitness levels, and the presence of comorbid conditions such as obesity or mental health disorders [24,25]. Additionally, methodological considerations such as exercise prescription parameters (intensity, frequency, duration), biological sample matrix (serum versus plasma), timing of sample collection, and population characteristics may contribute to the observed variability in BDNF responses [26,27].

Understanding the exercise-BDNF relationship in adolescents has significant clinical and public health implications, particularly given the rising prevalence of sedentary behavior, obesity, and mental health concerns in this population [28,29]. If exercise interventions can reliably modulate BDNF levels in adolescents, this knowledge could inform evidence-based strategies for optimizing cognitive development, academic performance, and overall brain health during this critical developmental period [30,31].

Given the limitations of previous reviews that included mixed study designs or focused on broader age ranges, and the critical need to establish causal relationships between exercise interventions and BDNF responses for clinical translation, the present systematic review specifically focused on randomized controlled trials—the gold standard for maximizing causal inference and minimizing bias when evaluating intervention efficacy. Therefore, this systematic review aimed to: (1) examine the effects of various exercise modalities on circulating BDNF concentrations in adolescent populations through exclusive analysis of randomized controlled trials, (2) evaluate the methodological quality of available studies, and (3) identify optimal intervention parameters to guide future research and evidence-based clinical practice.

## 2. Materials and Methods

### 2.1. Experimental Approach to the Problem

The present systematic review was conducted in accordance with the Preferred Reporting Items for Systematic Reviews and Meta-Analyses (PRISMA) guidelines (Supplementary Materials S1 and S2) [32] and adhered to established guidelines for conducting systematic reviews within the domain of sport sciences [33]. The review protocol was developed with the objective of ensuring comprehensive coverage of the relevant literature while maintaining methodological rigor. This systematic review was registered in PROSPERO (CRD420251071792).

### 2.2. Information Sources

The following bibliographic databases were consulted: FECYT (Web of Sciences, CCC, CIDW, KJD, MEDLINE, RSCI, and SCIELO), PubMed, SPORTDiscus, ProQuest Central, SCOPUS, and Cochrane Library. The search encompassed all published literature prior to 10 June 2025. The combination of databases was selected to ensure broad coverage of both medical and sports science literature.

### 2.3. Search Strategy

The PICO (Patient, Problem, or Population—Intervention or Exposure—Comparison, Control, or Comparator—Outcome[s]) framework was implemented to structure the search strategy and ensure systematic coverage of relevant literature. In the interest of maintaining transparency, the authors were not blinded to journal names or manuscript authors. The final search string was as follows:

(adolescent*) AND (exercise OR movement OR activity OR sport OR fitness OR aerobic OR training OR performance) AND (BDNF OR “brain-derived neurotrophic factor”) AND (“randomized controlled trial”)

### 2.4. Eligibility Criteria

The authors initiated the search string on databases and downloaded the title, authors’ names, journal, and date of all the articles that appeared in the search. Following the organization of the Excel spreadsheet, the process of removing all duplicates was initiated, and the remaining articles were subjected to a rigorous evaluation to ascertain their eligibility (Table 1).

### 2.5. Data Extraction

A standardized data extraction process was implemented using an Excel spreadsheet developed in accordance with the Cochrane Consumers and Communication Review Group’s data extraction template. The spreadsheet enabled a systematic evaluation of the inclusion and exclusion requirements for all the selected studies. The extraction process was conducted independently by two authors, with any disagreements being resolved through discussion until consensus was reached. Inter-rater agreement for study inclusion was assessed using Cohen’s kappa coefficient (≥0.80).

Extracted data included: study characteristics, participant demographics, intervention details (exercise modality, intensity, frequency, duration), BDNF measurement protocols (sample matrix, timing, assay method), and outcome measures. For incomplete data, corresponding authors were contacted via email with up to two follow-up attempts over four weeks. Non-responses were documented and considered in quality assessment. A full record was kept of all articles that were not included, including the particular reasons for exclusion.

### 2.6. Assessment of Study Methodology

The Physiotherapy Evidence Database (PEDro) scale was utilized to evaluate the methodological quality of pre-test post-test studies with experimental (EXP) and control (CON) groups that were randomly selected. The scale employs a range of 0 (low methodological quality) to 10 (high methodological quality) to score the internal study validity. The score that each section is awarded can range from 0 (“no”) to 1 (“yes”), depending on the quality obtained by each point. The quality of the studies were categorized according to the following cut-off points: excellent (9–10), good (6–8), fair (4–5), and poor (<3) [34]. The scale in question comprises ten items. Quality assessment was performed independently by two authors (A.M.-V. and M.R.-G.), with inter-rater reliability calculated using intraclass correlation coefficient (ICC ≥ 0.85). Disagreements were resolved through discussion, with third author (C.D.G.-C.) consultation if needed.

## 3. Results

**Figure 1:**
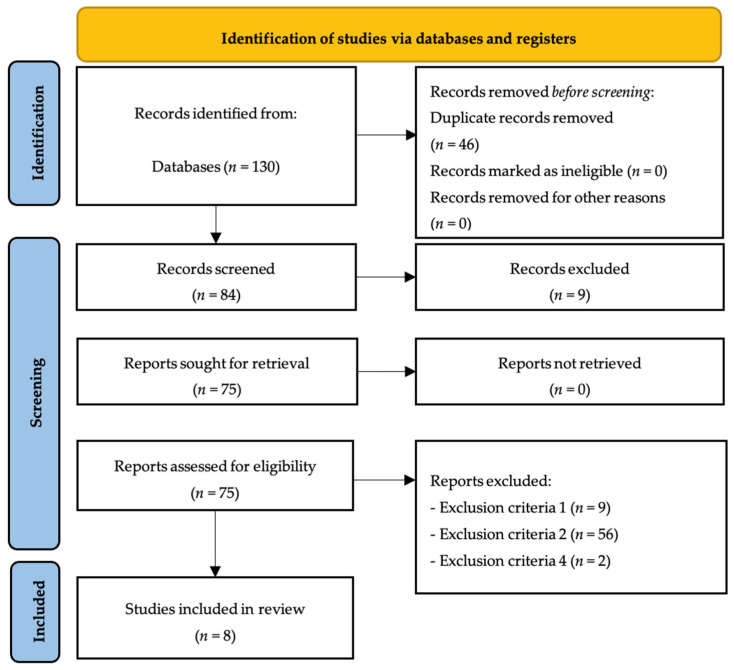
PRISMA flow diagram.

After analyzing all databases (FECYT: 5; PubMed: 5; SPORTDiscus: 1; ProQuest Central: 1; SCOPUS: 68; Cochrane Library: 46; external sources: 2), the contents of 130 articles were checked, detecting, at initial stage, 46 duplicate articles. Then, the authors analysed if each of the remaining 84 articles meet all inclusion criterion, resulting in the elimination of 74 articles by exclusion criteria number one (*n* = 9), exclusion criteria number two (*n* = 56) and exclusion criteria number four (*n* = 2). The remaining eight articles were included in the qualitative synthesis of the systematic review (Figure 1).

### 3.1. Methodological Quality

The quality assessment for this systematic review can be found in Table 2. Four of the eight studies included in this review were rated as good [18,20,21,35], whereas the remaining four were of fair quality [19,36,37,38]. Common methodological weaknesses included complete absence of subject and therapist blinding across all studies, which is understandable given the nature of exercise interventions. Assessor blinding was achieved in only four studies (50%) [20,21,35,38], and allocation concealment was poorly implemented, with only Wunram et al. [35] meeting this criterion. Follow-up rates were problematic, adequate retention above 85% was only achieved by two studies [18,19]. Notably, Seok-Min and Chol-Hyoung [37] demonstrated the poorest baseline group similarity reporting. All studies appropriately reported between-group statistical comparisons and provided point measures with variability measures.

### 3.2. Study Characteristics

The characteristics of included studies are presented in Table 3. Eight randomized clinical trials examining the effect of physical exercise on BDNF levels in adolescents were included, with substantial heterogeneity in methodological and population characteristics [18,19,20,21,35,36,37,38].

Sample sizes ranged from 18 to 304 participants, with ages spanning 12.55 to 18 years. Four studies included exclusively male participants [18,19,37,38], while others included mixed populations. Study populations were diverse, including healthy adolescents [18,19,38], adolescents with obesity [20,21,36,37], and those with major depressive disorder [35]. Most studies excluded participants with regular physical activity backgrounds [18,19,20,21,36].

Exercise modalities varied considerably, including aerobic training [18,19,38], resistance training [20,21], combined protocols [20,21,37], high-intensity interval training [19,38], and specialized disciplines such as Taekwondo [36] and whole-body vibration [35]. Intervention durations ranged from 6 weeks [35] to 24 weeks [20,21], with training frequencies between 3 and 5 sessions per week. Exercise intensities varied from 40% to 100% of maximum or reserve parameters.

All studies assessed BDNF levels using commercial ELISA kits, with seven studies analyzing serum samples [18,19,20,21,35,36,37] and one using plasma [38]. Sample collection protocols were consistent across studies, requiring 8–12 h fasting conditions and standardized timing to avoid acute exercise effects. Several studies incorporated complementary measures including growth factors [18,19,35], metabolic parameters [20,21], cognitive assessments [19,35,38], and oxidative stress markers [36].

### 3.3. Main Outcomes

Regarding the effects of exercise on BDNF levels, four studies showed statistically significant increases after intervention [18,19,35,36], while four studies found no significant changes [20,21,37,38]. Jeon and Ha [18] demonstrated a significant increase in serum BDNF levels following 8 weeks of moderate-intensity aerobic exercise in healthy male adolescents. In their subsequent study [19], the same authors reported intensity-dependent effects after 12 weeks of aerobic training, with significant increases observed in moderate-intensity and high-intensity groups, while low-intensity and control groups showed no significant changes.

Wunram et al. [35] found that 6 weeks of ergometer cycling training significantly increased BDNF levels compared to controls in adolescents with major depressive disorder. Whole-body vibration training showed a trend toward significance. Roh et al. [36] reported a significant time × group interaction after 16 weeks of Taekwondo training in overweight/obese adolescents, with the exercise group showing increased BDNF levels compared to controls.

Four studies failed to demonstrate significant BDNF changes. Walsh et al. [20] and Goldfield et al. [21] examined exercise interventions in adolescents with obesity and found no significant group × time interactions for BDNF after 24 weeks of aerobic, resistance, or combined training compared to diet-only controls. Gejl et al. [38] found no significant pre-post differences in plasma BDNF levels after 9 weeks of either high-intensity or moderate-intensity training in healthy adolescents. Similarly, Seok-Min and Chol-Hyoung [37] reported non-significant increases in BDNF after 12 weeks of combined aerobic and strength training in adolescents with obesity.

Studies reporting positive effects were predominantly conducted in male-only samples [18,19] or included specific clinical populations such as adolescents with depression [35] or overweight/obesity [36]. In contrast, studies with null findings included larger proportions of females [20,21] or examined healthy populations [38]. Exercise modalities varied from traditional aerobic training [18,19,38] to specialized activities such as Taekwondo [36], with intervention durations ranging from 6 weeks [35] to 24 weeks [20,21]. Additional variables examined across studies included oxidative stress markers, growth factors (IGF-1), metabolic parameters, and cognitive assessments. Notable secondary findings included associations between BDNF changes and improved glucose metabolism and beta cell function in adolescents with obesity [20].

### 3.4. Narrative Synthesis of BDNF Outcomes

A narrative synthesis of BDNF outcomes across the eight included studies revealed considerable heterogeneity in exercise-induced responses. Table 4 presents the pre- and post-intervention BDNF values, standard deviations, percentage changes, and *p*-values for each intervention group. (Table 4). The percentage changes in resting BDNF concentrations ranged from −4.51% to 27.7%, with four intervention groups achieving statistical significance. The synthesis identified three distinct response patterns: studies demonstrating significant BDNF increases, studies showing non-significant but notable changes, and studies reporting minimal alterations.

Studies showing significant BDNF increases were primarily those investigating exercise intensity effects and general fitness interventions. Jeon and Ha [19] demonstrated an intensity-dependent response pattern, with high-intensity exercise producing the largest increase (19.22%), moderate-intensity showing a modest but significant increase (6.99%), and low-intensity exhibiting minimal change (1.05%). Their subsequent investigation [18] confirmed exercise efficacy with an 18.19% increase compared to 3.53% in controls. Interestingly, Roh et al. [36] reported a paradoxical finding where the control group achieved significance (4.14% increase) while the exercise group, despite a larger magnitude increase (16.17%), did not reach statistical significance.

The remaining studies contributed to a pattern of non-significant responses across diverse populations and intervention types. Walsh et al. [20] examined participants with diabetes risk factors, finding minimal changes in both groups. Wunram et al. [35] investigated depression and cognitive function through three modalities: ergometer cycling (0.19% increase), whole-body vibration (0.33% decrease), and control conditions (1.42% increase). Gejl et al. [38] evaluated cardiorespiratory fitness interventions, with moderate-intensity training showing the largest single change across all studies (27.7%), though this remained non-significant. Goldfield et al. [21] compared exercise modalities in adolescents, revealing variable responses: aerobic training (0.42%), resistance training (2.05%), combined training (−4.51%), and controls (10.63%). Seok-Min and Chol-Hyoung [37] reported a 6.08% increase in exercise participants compared to minimal change (0.09%) in controls.

## 4. Discussion

This systematic review sought to analyze whether physical exercise can modulate circulating BDNF concentrations in adolescents. The search identified eight RCTs, each of good-to-excellent methodological quality, encompassing aerobic, resistance, concurrent, and high-intensity protocols, in addition to discipline-specific interventions such as Taekwondo and whole-body vibration. The resulting evidence may prove especially valuable to exercise professionals, healthcare practitioners, and school-based program designers interested in neurodevelopment and overall well-being in adolescents.

The main findings of this review yield mixed results. Although four trials demonstrated statistically significant post-intervention elevations in circulating BDNF [18,19,35,36], four detected no significant changes [20,21,37,38]. Collectively, these observations indicate that physical exercise is a plausible, though not definitive, modulator of BDNF in adolescents. The magnitude and direction of this effect likely hinge on exercise modality, dose (i.e., intensity, duration, and frequency), participant characteristics, and additional contextual factors.

Of the two studies that implemented resistance training [20,21], neither reported a significant post-intervention increase in BDNF. In this regard, other reviews on the effects of resistance training on BDNF levels in adults have also reported mixed findings. Babiarz et al. [39] found inconclusive evidence after analyzing ten studies on the effects of resistance training, with only four reporting positive results. Nevertheless, they concluded that intensities ≥ 70% of 1RM combined with short recovery periods are required to induce appreciable increases in BDNF. Similarly, the review by Huang et al. [40] found that five out of seven resistance training trials did not show significant changes in peripheral BDNF concentrations. The few reported increases were observed in studies lacking a control group.

Of the five included studies that implemented an aerobic exercise intervention, three reported statistically significant positive outcomes [18,19,35]. The trial conducted by Wunram et al. [35] demonstrated significantly greater improvements in adolescents with major depressive disorder following a six-week stationary cycling program. In contrast, previous research by Jeon and Ha [18,19] in obese adolescents reported significant increases in serum levels of BDNF after treadmill-based aerobic exercise (three to four sessions per week), supporting the hypothesis that the neurobiological benefits of exercise may be modulated by individual participant characteristics.

In this regard, available evidence in adults reveals mixed findings concerning BDNF responses to aerobic exercise. For instance, some short-term multi-session interventions (e.g., five weeks) have reported that only cognitive training, rather than structured aerobic exercise or mindfulness, induces sustained, modest increases in basal BDNF levels [41]. However, studies by Tsai et al. [42,43] have shown that while a single bout of moderate-intensity aerobic exercise can transiently elevate serum BDNF in young adults, these elevations do not significantly correlate with improvements in cognitive performance or neuroelectric indices (CNV and P3). Notably, only participants with higher cardiorespiratory fitness exhibit specific neurophysiological benefits, such as enhanced P3 amplitudes and reduced task-switching costs, suggesting that such effects may be driven more by fitness level than by BDNF per se. Thus, the mechanisms underlying exercise-induced cognitive enhancements may depend more on baseline physical fitness than on acute neurotrophic changes.

Therefore, several moderating factors may underlie the heterogeneous results observed across the included trials. Biological sex has been identified as a potential determinant of BDNF responsiveness to physical exercise [10,12], as estrogen is known to modulate both gene expression and peripheral release of this neurotrophin. However, most of the reviewed studies did not conduct sex-stratified analyses or adjust their statistical models accordingly, thereby limiting the interpretability of their findings. In addition, mental health status and body composition represent critical sources of variability. For instance, among adolescents with obesity, studies by Walsh et al. [20] and Goldfield et al. [21] found no significant changes in BDNF levels after aerobic, resistance, or combined training protocols. In contrast, Roh et al. [36] documented a significant increase in BDNF following a 16-week Taekwondo program in a similar population, suggesting that factors such as exercise modality, training intensity, and adherence may play a pivotal role in modulating neurotrophic responses. These discrepancies may reflect differences in exercise dose, baseline health status, initial fitness levels, or the influence of concomitant pharmacological treatments. Previous studies have suggested that session duration may influence acute BDNF levels [12]. On the other hand, during adolescence, the pubertal activation of the hormonal axis introduces additional complexity in the regulation of BDNF. Iughetti et al. [44] observed that boys in puberty exhibit significant lower plasma BDNF levels compared to their prepubertal peers and girls of similar age, suggesting a potential androgen-mediated modulation. Therefore, future RCTs should rigorously control for confounding variables such as sex, pubertal stage, menstrual cycle phase, and body composition, among others.

Despite the widespread use of commercial ELISA kits, seven studies analyzed serum samples [18,19,20,21,35,36,37] and only one utilized plasma [38]. This distinction may be relevant, as previous research by Tarassova et al. [26] highlighted that moderate-intensity exercise can increase plasma BDNF levels in older adults by up to 222%, whereas serum BDNF levels show only an 18% rise, with the effect dissipating within 35 min of rest. During the coagulation process, platelet activation induces the release of BDNF into serum [45], suggesting that clotting duration constitutes a critical methodological factor when quantifying serum BDNF concentrations [27]. Moreover, recent meta-analyses demonstrate an acute rise in BDNF levels after sessions of structured physical exercise, whereas long-term training programs do not increase circulating BDNF concentrations [46,47]. Additionally, extending the blood coagulation time to 30–60 min can substantially elevate BDNF levels [48]. In this regard, among the studies included in this review, blood sample collection occurred between 5 min and 72 h after the final exercise session. Given that BDNF typically returns to baseline within a few hours [49,50], such heterogeneous washout periods may have masked potential chronic adaptations. Therefore, future investigations should adopt standardized protocols for blood sample timing and handling to ensure the comparability and interpretability of findings.

Furthermore, although the present study has focused on BDNF levels as the primary variable, it is important to contextualize our findings in relation to the clinical effects observed in other secondary outcomes. For example, Jeon and Ha [19] reported a significant improvement in working memory. Numerous RCTs have consistently demonstrated improvements in cognitive function [51], academic performance [52], and mental health [53] in adolescents following physical exercise interventions.

Other studies, such as that by Walsh et al. [20], have shown that physical exercise interventions can improve glucose levels and HOMA-B indices, in line with previous findings [54]. Similarly, Seok-Min and Chol-Hyoung [37], Roh et al. [36], and Gejl et al. [38] observed significant improvements in body composition and physical fitness variables in adolescents. These results are consistent with previous systematic reviews suggesting that structured exercise programs are effective strategies for improving these outcomes in this population [55,56]. Therefore, physical exercise not only influences BDNF levels but may also provide adolescents with additional benefits in outcomes closely linked to their quality of life and overall functional capacity.

Although this original review includes high-quality and excellent RCTs, several limitations must be considered. First, the number of included studies was small, with limited sample sizes and a general lack of sex-stratified analyses. Second, a meta-analysis was not performed due to substantial heterogeneity in both study populations and interventions. Participants ranged from healthy individuals to those with obesity, depression, or anxiety, and the exercise protocols and control conditions varied widely, limiting the validity of pooled comparisons. Third, the timing of post-exercise sampling varied greatly across studies, which is a critical factor influencing BDNF levels and complicates cross-study comparisons. Finally, the adolescents assessed presented divergent health profiles (healthy, obese, and depressed), a variability that weakens external validity and further precludes meta-analysis.

## 5. Conclusions and Practical Applications

The available evidence from RCT indicates that exercise interventions may modulate peripheral BDNF levels in adolescents, though the effects are inconsistent across studies. Of the eight high-quality trials examined, only four demonstrated statistically significant increases in circulating BDNF concentrations following exercise interventions.

For exercise professionals and healthcare practitioners working with adolescents, these findings suggest several practical considerations for program design:

- Exercise interventions involving moderate-to-vigorous intensity activities.
- These interventions should be performed at least 2–3 times per week over a minimum period of 6 weeks.
- Multi-modal training approaches that integrate metabolic stress with cognitive and coordinative demands may be particularly effective.
- Effective examples may include martial arts, circuit training, and sport-specific activities.

## Figures and Tables

**Table 1:** Inclusion and exclusion criteria.

Item	Inclusion	Exclusion	Search Coherence
***Population***	Studies that include adolescents	Studies that do not include adolescents	adolescent
***Intervention or Exposure***	Adolescents doing exercise or physical activity	Adolescents not doing exercise or physical activity. Interventions where other factor is implemented (e.g., supplementation, transcranial stimulation) Study protocols Adolescents receiving pharmacological treatments	exercise OR movement OR activity OR sport OR fitness OR aerobic OR training OR performance
***Comparation***	-	-	
***Outcome[s]***	Outcomes related to brain-derived neurotrophic factor	Outcomes not related to brain-derived neurotrophic factor	BDNF OR “brain-derived neurotrophic factor”
***Design***	Randomized controlled trial	Non randomized controlled trials	“randomized controlled trial”
***Other criteria***	Peer-reviewed full-text studies published in original journal articles	Non-peer reviewed journal articles. Non original full-text studies (conference papers…).	

**Table 2:** Methodological assessment of the included studies.

	Jeon and Ha [18]	Jeon and Ha [19]	Walsh et al. [20]	Goldfield et al. [21]	Wunram et al. [35]	Roh et al. [36]	Seok-Min and Chol-Hyoung [37]	Gejl et al. [38]
Subjects were randomly allocated to groups	1	1	1	1	1	1	1	1
Allocation was concealed	0	0	0	0	1	0	0	0
The groups were similar at baseline regarding the most important prognostic indicators	1	1	1	1	1	1	0	1
There was blinding of all subjects	0	0	0	0	0	0	0	0
There was blinding of all therapists who administered the therapy	0	0	0	0	0	0	0	0
There was blinding of all assessors who measured at least one key outcome	0	0	1	1	1	0	0	1
Measures of at least one key outcome were obtained from more than 85% of the subjects initially allocated to groups	1	1	0	0	0	0	0	0
All subjects for whom outcome measures were available received the treatment or control condition as allocated or, where this was not the case, data for at least one key outcome was analyzed by “intention to treat”	1	0	1	1	1	0	1	0
The results of between-group statistical comparisons are reported for at least one key outcome	1	1	1	1	1	1	1	1
The study provides both point measures and measures of variability for at least one key outcome.	1	1	1	1	1	1	1	1
SCORE	6	5	6	6	7	4	4	5
CUT-OFF POINTS	Good	Fair	Good	Good	Good	Fair	Fair	Fair

**Table 3:** Main characteristics and findings about the effects of exercise on adolescents’ brain-derived neurotrophic factor.

Ref.	Participants	BDNF Registration	Other Criteria to Consider	Exercise Information	Results	Conclusions
Jeon and Ha [19]	*n* = 40 **Age:** 15.05 ± 0.78 years **Groups** ○ LIEG (*n* = 10): 15.06 ± 0.73 years ○ MIEG (*n* = 10): 15.47 ± 0.78 years ○ HIEG (*n* = 10): 15.15 ± 0.33 years ○ SG (*n* = 10): 15.05 ± 0.41 years **Country:** South Korea **Inclusion:** Male middle school students with no history of physical	**Biological matrix:** Serum. **ELISA method** ELISA Kit (Promega, Madison, WI, USA) using sandwich enzyme-linked immunosorbent assay **Sample collection:** 12-h overnight fasting, before and after 12 weeks of intervention. Whole blood centrifuged at 3000 rpm for 15 min, stored at −80 °C until analysis **Other variables:** IGF-1 (RIA, Toshiba YBA-200, Tokyo, Japan), Cortisol (RIA, Siemens, Munich, Germany).	All subjects abstained from any other physical activity during experimental period with school cooperation K-WISC-III working memory test (Korean version of Wechsler Intelligence Scale for Children-III, number subtest) for pre- and post-intervention assessment. 32 questions total with forward and reverse digit span tasks Post-intervention samples taken after one day of rest from exercise termination to avoid acute effects	**Duration and frequency:** 12 weeks (4 sessions/week) **Exercise groups:** Aerobic treadmill exercise at different VO_2_R intensities based on ACSM guidelines: ○ LI (Low Intensity): 40% VO2R ○ MI (Moderate Intensity): 55% VO2R ○ HI (High Intensity): 70% VO2R **Control group:** whole-body stretching for 30 min **Standardization:** Each session individually prescribed to achieve exactly 200 kcal energy expenditure	**BDNF changes** Significant differences in pre- to post-intervention BDNF values: ○ MI group: 25,900 ± 26,590 → 27,710 ± 25,860 pg/mL (*p* < 0.05) ○ HI group: 25,240 ± 34,170 → 30,090 ± 48,000 pg/mL (*p* < 0.05) ○ LI group: No significant change. ○ Control: No significant change. **IGF-1 results** No significant differences (*p* > 0.05) in pre- to post-intervention IGF-1 values in any intervention group. **Working memory** Significant improvements in working memory scores for HI group compared to other groups	Moderate- and high-intensity aerobic exercise induced significant increases in resting BDNF levels after 12 weeks The increase in BDNF was intensity-dependent, with greater effects observed in protocols ≥ 70% VO_2_R Long-term aerobic exercise of moderate to high intensity may have positive effects on serum BDNF concentrations at rest and on cognitive functioning in adolescents whose brains are still developing Exercise intensity appears to be a critical factor in determining BDNF response to chronic aerobic training
Walsh et al. [20]	*n* = 304 at baseline (*n* = 202 at 6-months) **Age:** 14–18 years (mean 15.4 ± 1.4 years) **Sex:** 70% females (136 girls, 66 boys) **Country:** Canada (Ottawa) **Groups:** ○ Exercise groups combined (*n* = 152) ○ Diet-only control (*n* = 50) **Inclusion:** Post-pubertal (Tanner IV–V) adolescents with BMI ≥ 95th percentile for age/sex OR ≥ 85th percentile with additional diabetes/cardiovascular risk factor.	**Biological matrix:** Serum **ELISA method:** Human Free BDNF Quantikine ELISA kit (R&D Systems, Minneapolis, MN, USA) **Sample collection:** 12-h overnight fasting, ~20 mL venous blood from forearm/antecubital vein. Baseline (before run-in) and at least 48 h after last exercise esión at 6-months to avoid acute effects. Stored at −80 °C **Sensitivity:** 20 pg/mL (range: 62.5–4000 pg/mL) **Other variables:** Fasting glucose (mmol/L), fasting insulin (mmol/L), HbA1c (%), HOMA-B (beta cell secretory capacity), HOMA-IS (insulin sensitivity), body composition via MRI	Exclusion of regular physical activity >2×/week for >20 min/session in 4 months prior At least 48 h after last exercise session to avoid acute effects 4 weeks supervised low-intensity exercise (aerobic + resistance), 4✕/week, required >80% adherence (13/16 sessions)	**Duration and frequency:** 24 weeks (4 sessions/week) **Exercise groups** ○ **Aerobic:** Treadmills, elliptical, bikes, 20 → 45 min, 65% → 85% HRmax ○ **Resistance:** 7 exercises, 20 → 45 min, 2 × 15 reps → 3 × 8 reps at 8-RM ○ **Combined:** Full aerobic + resistance programs **Control group:** Diet counseling only (250 kcal/day deficit) **For analysis:** Exercise groups combined vs. control.	**BDNF changes** ○ No significant group × time interaction for BDNF (*F*(3.227) = 0.89, *p* = 0.45) ○ No baseline associations between BDNF and anthropometric variables (all *p* > 0.33) ○ Control: No significant associations. **Significant results in exercise group** ○ Δ glucose vs. BDNF: *r* = −0.17, *p* = 0.04 ○ Δ HOMA-B vs. BDNF: *r* = 0.23, *p* = 0.005 **Adjusted regression (exercise group)** ○ Change in HOMA-B predicting BDNF: *β* = 0.093, *SE* = 0.031, *p* = 0.004	Exercise-induced BDNF changes were associated with improvements in fasting glucose and beta cell function (HOMA-B) in adolescents with obesity First pediatric study to demonstrate associations between BDNF and beta cell function following exercise intervention HOMA-B is one of the strongest predictors of type 2 diabetes development, making this finding clinically significant Exercise may work through both direct metabolic effects and indirect BDNF-mediated pathways to improve diabetes risk
Wunram et al. [35]	*n* = 64 **Age:** 15.88 ± 1.15 years **Sex:** 18 males and 46 females **Country:** Germany **Groups** ○ Ergometer Cycling (EC) (*n* = 20) ○ Whole-Body-Vibration (WBV) (*n* = 21) ○ Treatment as Usual (TAU) control (*n* = 23) **Inclusion:** Major depressive disorder (MDD) diagnosed with SKID-I, DIKJ score ≥ 18, IQ > 70. Anxiety disorders (*n* = 14), somatoform (*n* = 8)	**Biological matrix:** Serum. **ELISA method:** Quantikine^®^ Human BDNF kit (R&D Systems) **Sample collection:** Fasting blood samples at T0 (baseline), T1 (after 6 weeks) and T2 (after 14 weeks). **Other variables:** IGF-1 and p.Val66 polymorphism of the BDNF gene.	Drug treatment allowed under specific conditions (stable medication < 3 weeks). Usual therapy maintained in all groups. Factors controlled were: BMI percentiles, number of sessions, additional sport, medication, age, sex and genotype.	6 weeks of intervention (3/5 times a week) + 8 weeks of follow-up with no exercise **Exercise group (30 min per session)** ○ **EC:** 7-step interval (40%-50%-70%-50%-70%-50%-40%) with an cadence 60–70 rpm and a progression of 70% → 80% after 12 h sessions ○ **WBV:** 6 exercises, 2 → 3 min each with 20 Hz frequency and 2-cm amplitude with 2 → 3 min rest between exercises **Control group:** usual treatment including single- and group psychotherapy, art, music, and conventional sports therapy	**BDNF changes** ○ EC group: significantly higher vs. controls over entire period (6860 ± 2731 pg/mL, *p* = 0.016) ○ WBV group: trend vs. controls (4852 ± 2704 pg/mL, *p* = 0.079) ○ Both groups higher than controls after 6 weeks ○ EC maintained significance at 14 weeks **IGF-1 changes** ○ EC group: significant increase at t1, decreased during rest period. ○ WBV and controls: no significant changes. **Genetic analysis** Val66Val variant showed trend for better exercise response.	Endurance exercise (EC) showed superior effects on BDNF and IGF-1 compared to muscle strengthening (WBV) Both interventions influenced peripheral neurotrophins in depressed adolescents Changes in growth factors did not correlate with depression score improvements BDNF p.Val66Val variant may be more receptive to exercise treatment Biomarkers could help develop tailored treatment strategies for adolescent depression
Gejl et al. [38]	*n* = 85 completed **Age:** 16–19 years (females: 17.8 ± 0.8, males: 18.0 ± 0.9) **Sex:** 58 females and 27 males **Country:** Denmark **Groups** ○ High-Intensity Training (HIT) (*n* = 27) ○ Moderate-Intensity Training (MIT) (*n* = 23) ○ Control (CON) (*n* = 35) **Inclusion:** Untrained or recreationally active high school adolescents	**Biological matrix:** Plasma. **ELISA method:** BDNF kit (R&D Systems) **Sample collection:** Fasting morning collection (>8 h), before and after 9-week intervention, venous blood with EDTA.	Sociodemographic variables and sex were adjusted for in the analyses. Cognitive tests (flanker task) and measures of VO_2_max were also part of the protocol.	9 weeks (3 sessions/week) **Exercise groups (30 min/session, combination of cycling and running)** ○ **HIT:** 80–100% heart rate reserve (HRR) ○ **MIT:** 60–70% HRR **Control group:** Continued habitual lifestyle.	**BDNF changes** ○ No significant differences in pre-post in either intervention group (HIT: From 274–407 pg/mL to 261–475 pg/mL; *p* = 0.731; MIT: From 266–345 pg/mL to 314–471 pg/mL; *p* = 0.055). ○ There was no significant difference in changes between groups (Χ2 = 1.662; *p* = 0.436). **VO_2_max changes** ○ HIT: Larger increase vs. both CON and MIT groups ○ MIT: Modest improvements ○ CON: No significant changes	High-intensity training was superior for improving cardiorespiratory fitness but had no effect on plasma BDNF levels Neither training intensity influenced inhibitory control or BDNF in healthy adolescents Results contrast with previous studies showing BDNF increases with exercise in adolescents Null findings may be related to population characteristics (healthy vs. clinical populations) or methodological factors
Goldfield et al. [21]	*n* = 282 at baseline **Age:** 14–18 years (15.6 ± 1.4 years) **Sex:** 197 females and 85 males. **Country:** Canada **Groups** ○ Aerobic training (*n* = 69) ○ Resistance training (*n* = 70) ○ Combined (*n* = 74) ○ Non-exercising control (*n* = 76) **Inclusion:** Post-pubertal (Tanner IV–V) adolescents with BMI ≥ 95th percentile for age/sex OR ≥ 85th percentile with additional diabetes/cardiovascular risk factor	**Biological matrix:** Serum. **ELISA method:** (Human Free BDNF Quantikine®, R&D Systems, Cat# DBD00); duplicate; dilution 1:75. **Sample collection:** 12-h overnight fasting, ~20 mL venous blood from forearm/antecubital vein. Baseline (before run-in) and 2–10 days after last exercise session at 6-months to avoid acute effects. Stored at −80 °C **Sensitivity:** 20 pg/mL (range: 62.5–4000 pg/mL)	Exclusion of regular physical activity >2✕/week for >20 min/session in 4 months prior. Physical activity from school PE classes was not an exclusion criterion Stable medication doses required for 2 months prior and throughout trial 4 weeks supervised moderate-intensity exercise (aerobic + resistance), 4✕/week, required >80% adherence (13/16 sessions)	24 weeks (4 sessions/week) **Exercise groups** ○ Aerobic: Treadmills, elliptical, bikes, progressive 20 → 45 min, 70–85% VO2 peak ○ Resistance: 7 exercises, progressive 20 → 45 min, ≥1 min breaks between sets ○ Combined: Progressive to 90 min (45 min aerobic + 45 min resistance) **Control group:** Diet counseling only (250 kcal/day deficit)	**BDNF changes** ○ No significant group × time interaction (F(3,227) = 0.89, *p* = 0.45) ○ No main effect of time (*F*(1,227) = 0.01, *p* = 0.91) ○ No main effect of group (*F*(3,250) = 0.74, *p* = 0.53) **Intention-to-treat BDNF changes (ng/mL, baseline → 6-months):** ○ Aerobic: 24.6 → 26.4 (change: 1.8, *p* = 0.44) ○ Resistance: 29.7 → 27.7 (change: −1.9, *p* = 0.44) ○ Combined: 27.9 → 26.2 (change: −1.7, *p* = 0.46) ○ Control: 25.8 → 28.1 (change: 2.3, *p* = 0.33) **Per-protocol analysis (≥70% adherence):** Similar null findings (all *p* > 0.05)	Aerobic training, resistance training, or their combination did not change serum BDNF levels in adolescents with obesity over 6 months BDNF responses to exercise are heterogeneous and highly variable, with >60% of adult studies showing null findings Sex differences may explain discrepant findings compared to previous male-only studies that showed BDNF increases
Roh et al. [36]	*n* = 20 **Age:** 12.55 ± 0.51 years **Sex:** 14 males and 6 females **Country:** South Korea **Groups** ○ Experimental group (EG, *n* = 10) ○ Control group (CG, *n* = 10) **Inclusion:** Overweight and obese adolescents with BMI ≥ 85th percentile for age/sex based on 2007 Korean growth standards	**Biological matrix:** Serum. **ELISA method:** (R&D Systems, Cat# DY248). Collection of 10 mL fasting blood (8 h), centrifuged and stored at −80 °C; absorbance analysis at 450 nm in spectrophotometer (Tecan Sunrise, TECAN, Grödig, Austria). **Other variables:** Oxidative stress (MDA, SOD), myokines (IL-15, irisin, myostatin), physical fitness (VO_2_max, grip strength, leg strength, sit-and-reach, Sargent jump, stork stand test)	Not participating in other regular exercise programs except school PE; no experience of Taekwondo training; no musculoskeletal disease; not taking growth medications	16 weeks (5 sessions/week) **Exercise group:** Taekwondo technique training as aerobic training. **Control group:** No treatment (maintained normal activities)	**BDNF changes** ○ Significant time × group interaction (F = 6.767, *p* = 0.018) ○ EG: 25.41 ± 5.36 → 29.52 ± 5.83 (significant increase, *p* < 0.05) ○ CG: 26.58 ± 6.10 → 27.68 ± 6.50 (no significant change, *p* > 0.05) **Significant results in other variables:** ○ SOD: Higher in EG post-intervention vs. CG (*p* < 0.05) ○ MDA: Lower in EG post-intervention vs. CG (*p* < 0.05) ○ Irisin: Significantly decreased in EG (*p* < 0.05) ○ Physical fitness: Improved leg strength, flexibility, power in EG (*p* < 0.05)	16 weeks of Taekwondo increases serum BDNF levels in overweight/obese adolescents. Regular Taekwondo training could be an effective strategy to improve neurotrophic profile and mitigate oxidative stress in overweight pediatric population.
Jeon and Ha [18]	*n* = 20 **Age:** 15 years **Sex:** 100% males **Country:** South Korea **Groups:** ○ Exercise group (EG, *n* = 10) ○ Control group (CG, *n* = 10) **Inclusion:** Healthy junior high school students with no history of physical illness	**Biological matrix:** Serum. **ELISA method**: ELISA kit (sandwich enzyme-linked immunosorbent assay), Promega, USA **Sample collection:** Blood samples obtained 2 days before and 2 days after exercise intervention. Whole blood centrifuged at 3000 rpm for 15 min, stored −80°. **Other variables:** IGF-1 (ng/mL), cortisol (μg/dL)	All participants were asked to maintain their usual diet and activity level during the study. Students excluded if taking part in sports activities beyond usual school PE curriculum; asked not to undertake exercise other than study protocol	8 weeks (3 sessions/week) **Exercise group:** Aerobic treadmill: 40–60% of VO_2_R (oxygen reserve), according to ACSM recommendations. **Control group:** Continued normal sedentary activities Each session individually adjusted to ensure an energy expenditure of 200 kcal.	**BDNF changes** ○ Significant increase in exercise group (*p* < 0.01) ○ EG: 23.32 ± 6.25→ 27.57 ± 5.65 (*p* < 0.01) ○ CG: 24,071.3 ± 6139.2 → (*p* > 0.05) ○ Significant between-group differences in BDNF after 8 weeks (*p* < 0.01) **Significant results in other variables:** ○ IGF-1: Significant increase in EG (397.1 ± 131.1 → 432.3 ± 165.2 ng/mL, *p* < 0.05) ○ Cortisol: Non-significant decrease in EG (13.6 ± 4.1 → 11.2 ± 2.3 μg/dL, *p* > 0.05)	Long-term regular aerobic exercise has positive effects on enhancement of BDNF levels at rest in adolescents undergoing brain development 8 weeks of chronic aerobic exercise significantly increased both BDNF and IGF-1 expression in adolescents Exercise intensity of 40–60% VO_2_R was sufficient to elicit significant BDNF increases when standardized for energy expenditure.
Seok-Min and Chol-Hyoung [37]	*n* = 18 **Age:** 12.78–13 ± 0.71–0.83 years **Sex:** 100% males **Country:** South Korea **Groups:** ○ Exercise group (EG, *n* = 9) ○ Control group (CG, *n* = 9) **Inclusion:** With obesity IMC ≥ 85% percentile	**Biological matrix:** Serum. **ELISA method:** (Ab Frontier Human BDNF ELISA Kit, Catalog # LF-EK5005, Seoul, Republic of Korea) **Collection time:** Fasting (≥10 h), at 8:00 a.m., EDTA-treated venous extraction; storage at −70 °C. **Other variables:** Total cholesterol (TC), LDL-cholesterol (LDL-C), glucose, body composition parameters (weight, BMI, %fat, fat mass, %LBM)	Participants avoided intense exercise 12 h before the measurements and fasted 4 h before the DXA test. Lipid profile and body composition were monitored by DXA.	12 weeks (3 sessions/week) **Exercise group** Concurrent training: ○ Aerobic: 65–75% HRmax ○ Strength: 60–70% 1RM × 10–15 reps × 3 sets **Control group:** Normal sedentary activities	**BDNF changes** ○ Non-significant increase in exercise group (*p* > 0.05) ○ EG: 33.70 ± 4.14 → 35.75 ± 2.83 ng/mL (diff: +2.06 ± 4.1) ○ CG: 33.18 ± 3.69 → 33.21 ± 2.94 ng/mL (diff: +0.03 ± 4.9) **Significant results in other variables:** ○ Improvements of the exercise group in % body fat (34.27 ± 4.24% to 31.52 ± 4.07%) and % fat free mass (62.49 ± 4.21% to 65.17 ± 3.95%).	The 12-week combined exercise program significantly improved body composition parameters, but did not produce statistically significant changes in BDNF. The combination of aerobic and strength training may be effective for weight control and improvement of body composition in adolescents with obesity, although its effect on BDNF requires further evidence.

**Note:** *p*, significance; F, F-statistic from ANOVA tests; r, Pearson correlation coefficient; β, beta coefficient in regression analysis; SE, standard error of the mean; Χ^2^, chi-square statistic; diff, difference between pre- and post-intervention values; →, indicates change from pre-intervention to post-intervention values; N, total sample size; EG, Exercise Group; CG, Control Group; LIEG, Low Intensity Exercise Group; MIEG, Moderate Intensity Exercise Group; HIEG, High Intensity Exercise Group; SG, Stretching Group (control); EC, Ergometer Cycling group; WBV, Whole-Body Vibration group; TAU, Treatment as Usual (control); HIT, High-Intensity Training; MIT, Moderate-Intensity Training; CON, Control group; BDNF, Brain-Derived Neurotrophic Factor; IGF-1, Insulin-like Growth Factor 1; VO_2_R, oxygen uptake reserve (percentage of reserve capacity); VO_2_ peak/max, peak/maximal oxygen consumption; HRmax, maximum heart rate; HRR, heart rate reserve; 1RM, one repetition maximum (strength testing); BMI, Body Mass Index; %LBM, percentage lean body mass; MDA, malondialdehyde (oxidative stress marker); SOD, superoxide dismutase (antioxidant enzyme); ELISA, Enzyme-Linked Immunosorbent Assay; RIA, radioimmunoassay; EDTA, ethylenediaminetetraacetic acid (anticoagulant); DXA, dual-energy X-ray absorptiometry; MRI, Magnetic Resonance Imaging; K-WISC-III, Korean version of Wechsler Intelligence Scale for Children-III; SKID-I, Structured Clinical Interview for DSM-IV Axis I Disorders; DIKJ, Depression Inventory for Children and Adolescents (German); HOMA-B, Homeostatic Model Assessment of Beta-cell function; HOMA-IS, Homeostatic Model Assessment of Insulin Sensitivity; HbA1c, glycated hemoglobin (average blood glucose over 2–3 months); TC, Total Cholesterol; LDL-C, Low-Density Lipoprotein Cholesterol; HDL-C, High-Density Lipoprotein Cholesterol; TG, triglycerides; MDD, Major Depressive Disorder; Val66Val, Valine-Valine genotype of BDNF gene polymorphism; Val66Met, Valine-Methionine genotype of BDNF gene polymorphism; pg/mL, picograms per milliliter (1 ng/mL = 1000 pg/mL); ng/mL, nanograms per milliliter; μg/dL, micrograms per deciliter; mmol/L, millimoles per liter; ACSM, American College of Sports Medicine; PE, Physical Education.

**Table 4:** Summary of pre-post BDNF values, percentage changes, and statistical significance across intervention groups.

Study	Group	Pre	Post	SD_Pre	SD_Post	Δ%	*p*	Clinical Outcomes	
								Primary	Secondary
Jeon and Ha [19]	LIEG	24.79	25.05	25.77	21.47	1.05	>0.05	Working memory performance (K-WISC-III number subtest, raw scores)	IGF-1, Cortisol, BDNF
	MIEG	25.90	27.71	26.59	25.86	6.99	<0.05		
	HIEG	25.24	30.09	34.17	48.00	19.22	<0.01		
	Control	23.96	24.50	20.93	22.04	2.25	>0.05		
Walsh et al. [20]	Exercise	26.2	–	14.3	–	–	>0.05	Diabetes risk factors (glucose, insulin, HOMA-B, HOMA-IS, HbA1c), body composition	BDNF
	Control	25.2	–	14	–	–	>0.05		
Wunram et al. [35]	EC	35.92	35.99	10.91	–	0.19	0.75	Depression (DIKJ, BDI-II)	Cognitive function, IGF-1, BDNF
	WBV	33.12	33.01	10.36	–	−0.33	0.64		
	Control	29.48	29.90	7.51	–	1.42	0.12		
Gejl et al. [38]	HIT	33.4	34.7	–	–	0.89	0.73	Cardiorespiratory fitness (VO_2_max), inhibitory control (flanker task)	BDNF, resting heart rate
	MIT	29.2	37.3	–	–	27.7	0.06		
	Control	30.4	30.0	–	–	−1.31	0.89		
Goldfield et al. [21]	Aerobic	23.7	23.8	2.5	2.5	0.42	0.98	Body composition (DXA)	BDNF, mental health, cardiorespiratory fitness (VO_2_max)
	Resistance	24.4	24.9	3.1	3.2	2.05	0.92		
	Combined	28.8	27.5	2.7	2.8	−4.51	0.71		
	Control	25.4	28.1	2.0	2.1	10.63	0.30		
Roh et al. [36]	Exercise	25.41	29.52	5.36	5.83	16.17	<0.05	Physical fitness, anthropometrics	Oxidative stress biomarkers, myokines, BDNF
	Control	26.58	27.68	6.10	6.50	4.14	>0.05		
Jeon and Ha [18]	Exercise	23.32	27.57	6.25	5.65	18.19	<0.001	Physical fitness/exercise response	IGF-1, cortisol, BDNF
	Control	24.07	24.92	6.14	7.82	3.53	>0.05		
Seok-Min and Chol-Hyoung [37]	Exercise	33.70	35.75	4.14	2.83	6.08	>0.05	Body composition, blood lipids	BDNF, glucose
	Control	33.18	33.21	3.69	2.94	0.09	>0.05		

**Note.** BDNF = brain-derived neurotrophic factor; LIEG = low intensity exercise group; MIEG = moderate intensity exercise group; HIEG = high intensity exercise group; EC = ergometer cycling; WBV = whole-body vibration; HIT = high-intensity training; MIT = moderate-intensity training. Values are presented as mean BDNF concentrations in pg/mL. Pre and Post represent baseline and post-intervention values, respectively. Δ% represents percentage change calculated as ((Post-Pre)/Pre) × 100. A dash (–) indicates information not reported in the original study.

## Data Availability

No new data were created or analyzed in this study. Data sharing is not applicable to this article.

## Supplementary Materials

The following supporting information can be downloaded at: https://www.mdpi.com/article/10.3390/sports13080253/s1, File S1: The PRISMA 2020 statement: an updated guideline for reporting systematic reviews. File S2: The PRISMA for Abstracts Checklist.
